# The Effect of Age and Sex on the Rate of Germline Mutations in Barn Owls

**DOI:** 10.1093/gbe/evag175

**Published:** 2026-07-10

**Authors:** Alexandros Topaloudis, Anne-Lyse Ducrest, Ana Drago-Rosa, Céline Simon, Bettina Almasi, Alexandre Roulin, Jérôme Goudet

**Affiliations:** Department of Ecology and Evolution, University of Lausanne, Lausanne CH 1015, Switzerland; Swiss Institute of Bioinformatics, Lausanne 1011, Switzerland; Department of Ecology and Evolution, University of Lausanne, Lausanne CH 1015, Switzerland; Department of Ecology and Evolution, University of Lausanne, Lausanne CH 1015, Switzerland; Department of Ecology and Evolution, University of Lausanne, Lausanne CH 1015, Switzerland; Swiss Ornithological Institute, Sempach CH 6204, Switzerland; Department of Ecology and Evolution, University of Lausanne, Lausanne CH 1015, Switzerland; Department of Ecology and Evolution, University of Lausanne, Lausanne CH 1015, Switzerland; Swiss Institute of Bioinformatics, Lausanne 1011, Switzerland

**Keywords:** birds, trios, mutation spectrum, sex differences

## Abstract

The rate of new mutations is the ultimate source of genetic variation, and thus, a fundamental quantity in evolution. Mutation rates vary across the tree of life, influenced by selection pressures and life-history traits. Within species, mutation rates and types show differences between sexes as well as a positive association with parental age. Most of the direct estimates come from a few mammal species, often primates, and their generality has not been fully realized. Using 33 parent–offspring trios, we investigate the mutation rate in the barn owl (*Tyto alba*) and show that the species has a mutation rate of 4.96 × 10^−9^ per site per generation, matching rates of passerine species with a similar generation time of 2 to 3 years. Using read- and trio-based phasing methods we find that fathers pass on twice as many mutations as mothers and identify subtle differences in the sex-specific spectra of mutations. We also show that the number of mutations increases with paternal age, bringing direct evidence for germline senescence in birds. While across-species mutation rates and types appear conserved, the potential for within species variation due to age is substantial.

Significance The number of new mutations that arise on a genome is influenced by sex and age but most of what we know originates from mammals and mostly primates. Using 33 trios of barn owls we find a mutation rate as similarly lived passerines and an increased number of mutations from older fathers. Our work brings direct evidence of mutation rate dependence on age at reproduction in birds, highlighting how life-history can drive the rate of molecular evolution.

## Introduction

Understanding the rate at which mutations appear can determine both the burden of genetic disease and the pace of adaptation. New mutations arise due to mistakes in DNA replication or DNA lesions from mutagenic factors (Baer et al. 2007; Seplyarskiy and Sunyaev 2021), which become incorporated in the DNA sequence due to failure of repair mechanisms (Lindahl and Wood 1999). These errors are subsequently inherited by daughter cells, leading to the accumulation of mutations with increasing age of a cell lineage, a burden that can, for example, lead to cancer (Moore et al. 2021). The mutations that arise in the germline, the subset of cells that will eventually produce gametes, can be inherited by the next generation, introducing heritable variation on which selection can act, leading to evolution.

In the last two decades, accessible sequencing methods have enabled direct measurement of mutation rates through parent–offspring (trio) studies across a broad range of species (Keightley et al. 2014; Smeds et al. 2016; Pfeifer 2017; Bergeron et al. 2023; Suárez-Menéndez et al. 2023; Wang and Obbard 2023; Zhang et al. 2023; Armstrong et al. 2025). These studies have revealed that per-generation mutation rates can vary by a factor of 40 across animals. Among the factors influencing this diversity are life-history traits such as generation time and mating system, effective population size, and genome size (Lynch 2010; Bergeron et al. 2023; Wang and Obbard 2023; Weinstein and Roy 2026). However, so far, estimates of mutation rates for most clades are only represented by a few species, often in one or a few trios of captive individuals. Because the age of an individual and its captivity status can also influence the number of mutations, a more robust approach would involve sampling multiple families of different parental ages from individuals living in the wild. Such study designs can help determine within and between species variation and shed light into the forces driving variation in mutation rates.

Sex and age modulate the rate and type of new mutations introducing variation within species. In mammals and birds, there is a pronounced paternal bias in the mutations inherited by offspring (Kong et al. 2012; de Manuel et al. 2022; Bergeron et al. 2023). In addition, the number of mutations increases with increasing paternal (Kong et al. 2012; Venn et al. 2014; Thomas et al. 2018; Wang et al. 2020, 2022; Versoza et al. 2025) and maternal age (Goldmann et al. 2016; Rahbari et al. 2016). Starting at puberty, spermatogonia undergo continuous mitotic divisions, to produce sperm and replenish themselves. Continuous divisions can lead to accumulating mutations through replication errors although some of these mutations may be instead damage-induced (Gao et al. 2016, 2019; Spisak et al. 2024). On the contrary, maternal oocytes do not divide postnatally and the increase in mutations observed occurs exclusively through damage to the DNA. In addition, in humans, mutations from sperm and eggs have a different nucleotide context (mutational spectrum), due to the different origin of mutations or repair processes which might be sex-specific (Goldmann et al. 2016; Jónsson et al. 2017; Stringer et al. 2020). Most of these findings originate from large human datasets and confirmation of these patterns in other species is lacking.

Birds are a compelling class to study the process of germline mutation as they exhibit substantial variation in life-history traits, from short-lived, promiscuous passerines to long-lived, monogamous seabirds. In addition, birds show a lower male bias than mammals (de Manuel et al. 2022), but with considerable variation among species (Bergeron et al. 2023). Recent work on 18 species (Bergeron et al. 2023), found that avian mutation rates differ by 2 orders of magnitude, and that the snowy owl (*Bubo scandiacus*) exhibited the lowest mutation rate among all species analyzed (yearly rate of 1.6 × 10^−10^). While there is a strong effect of generation time among species, it remains unclear how the age of the parents influences the rate of germline mutations in birds. In addition, differences in the type of mutations from each sex have not been investigated.

We explore these questions using a direct estimate of the germline mutation rate in the Western barn owl (*Tyto alba*) based on 33 parent–offspring trios sequenced at high depth. This large data set, among the largest for any nonhuman vertebrate, enables us to robustly characterize both the average mutation rate and sex-specific mutation spectra as well as quantify effects of parental age on mutation accumulation. Our findings provide critical insights into the forces shaping germline mutation in a widespread avian predator and contribute to the broader understanding of mutation rate evolution across vertebrates.

## Results

### Mutation Rates

**Fig. 1.**
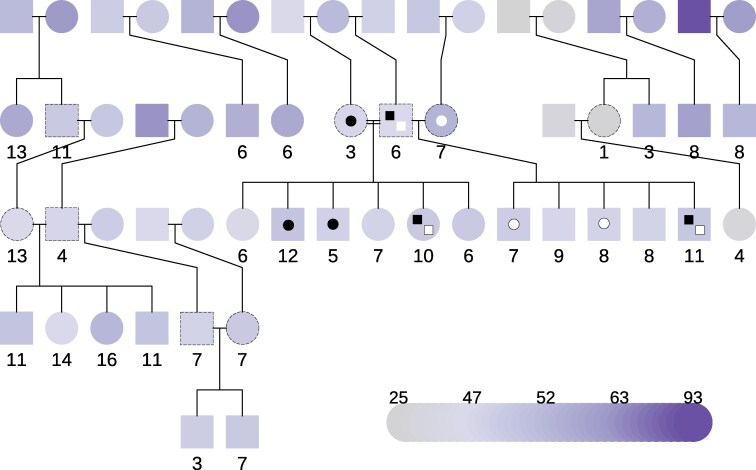
The dataset used in the study. The numbers are the counts of observed mutations after manual inspection. The color shows the average sequencing depth. The dashed outline individuals are those who have both parents and offspring in the dataset and the inheritance of mutations could be tested. The black and white symbols are mutations of maternal (circles) and paternal (squares) origin which are shared between siblings and the parent of origin is also signified accordingly.

Using 57 individuals in 33 trios, sequenced at an average sequencing depth of 54× (range: 25 to 93×; Fig. 1) and stringent filtering, we identified 255 forward (reference to alternative) and 3 reverse (alternative to reference) mutations as putative mutational events. These mutations were not present in the parents of the trio or in Swiss samples unrelated to the focal individual and are thus called de novo mutations (DNMs) hereafter. On average an offspring carried 7.8 DNMs (range 1 to 16; Fig. 1). The callable genome (CG) in each trio was on average 805 Mb (range 324 to 899) and was influenced by sequencing depth (Fig. S1). After applying a false discovery rate (FDR) correction (mean = 3.4%; range = 1.87 to 7.37), the average mutation rate per trio was 4.96 × 10^−9^ (95% CI: 4.24 × 10^−9^ to 5.68 × 10^−9^; range 1.66 × 10^−9^ to 9.8 × 10^−8^). The average age at reproduction in our dataset was 2.73 years bringing the yearly mutation rate to 1.82 × 10^−9^. In nine individuals (outlined in Fig. 1) where segregation of 59 candidate DNMs to the next generation could be tested, we found no evidence of non-Mendelian inheritance of these DNMs using a binomial test.

Mutations appeared in all 39 autosomal linkage groups (LGs), independently segregating molecules identified through linkage mapping (Topaloudis et al. 2025), except one (LG38, size = 7.6 Mb). The number of DNMs identified per LG correlated strongly with the number of callable sites per LG (Pearson's correlation, *r* = 0.7) which in turn scaled almost perfectly (*r* = 0.99) with a LGs physical length (Fig. S2). There was no difference between micro- (<20 Mb) and macro- (>20 Mb) chromosomes in the number of mutations (*β* = 0.6; SE = 1.4; *t* = 0.4; *P* = 0.69), once length was accounted for. On the Z chromosome, we found four mutations in two trios, none of which in the pseudoautosomal region. The per-trio mutation rate for the Z was 5.57 and 6.71 × 10^−8^ and the Z mutation rate among all trios tested was 4.97 × 10^−9^, within the range of the autosomal estimate.

Of the 258 DNMs (Table S1), four were shared between two siblings each, but were absent from the somatic tissue of the parents (Fig. 1). An explanation is that these mutations originated in the primordial germ cells (PGC) of the parents. All four of the putative PGC mutations were C to T transitions. Through read-based phasing we found two of them to be of paternal and two of them of maternal origin. These mutations made up 6%, 11%, and 14% of the total phased mutations originating from the focal parent(s) respectively. We retained these mutations in downstream analyses as they represent true mutation events that are passed on to the offspring and contribute to the generational mutation rate.

### Mutation Spectra

**Fig. 2.**
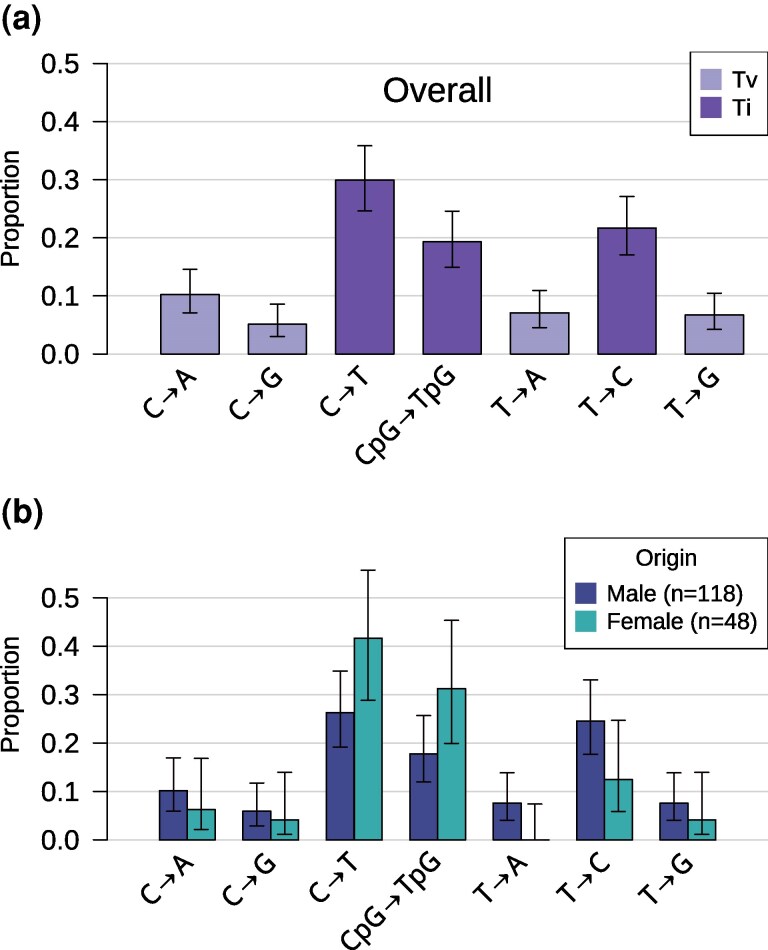
The de novo mutation spectrum. a) Overall mutation spectrum in the 254 unique mutations. b) The proportion of mutations of each class originating in each sex. Ti = transitions, Tv = transversions. Error bars show 95% binomial confidence intervals. Each category includes its reverse complement.

The mutation profile was skewed in favor of transitions (Ti) over transversions (Tv), with a ratio of Ti/Tv equal to 2.43 (Fig. 2). The most common class of mutations in the 258 unique identified DNMs, were C to T (including G to A) transitions which comprised 125 (49%) of the total DNMs observed. Of these, 49 (19% of the total) were in CpG dinucleotides. The second most common class was T to C transitions (22%) followed by transversions from C to A (10%). The spectrum was similar to those from multitrio studies in birds and the one estimated in humans (Fig. S3; Fisher's exact test *P* = 0.25) though each study used a different approach in mutation calling and filtering (Table S2).

Of the 254 unique DNMs we managed to phase 166 (66%). Of these 54 were phased by segregation to a third generation while 153 were phased using read-based phasing and nearby phase-informative sites in the focal trio. The average phasing success per trio was 65% (range 17% to 100%). Most of the phased mutations had a paternal origin (118 compared to 48 of maternal origin) bringing the male to female ratio (*α*) to 2.5. The overall mutation profile was significantly different between the sexes (Fisher's exact test with simulated *P* values: *P* = 0.034). Maternally inherited mutations tended to be overrepresented in the C to T mutation class, and especially in a CpG context. On the contrary, males passed on a larger number of T to C transitions. As a consequence, the Ti/Tv ratio in females was 5.86 compared to 2.19 in males. No individual class was significantly different after multiple testing correction.

### Effect of Age

**Fig. 3.**
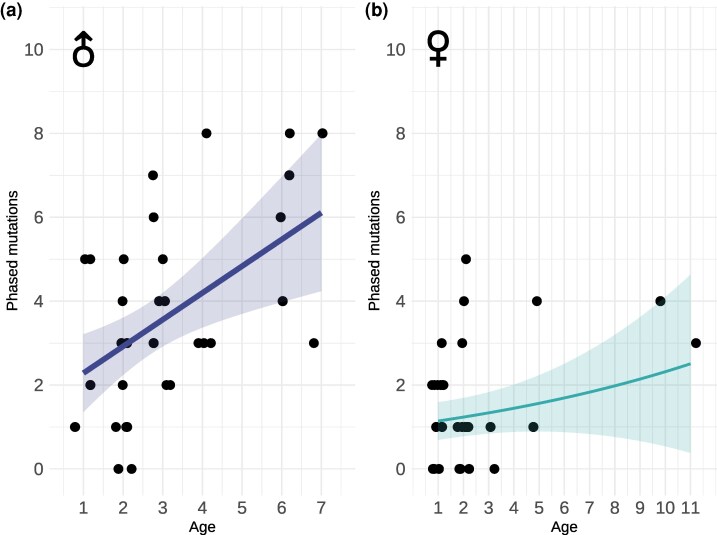
Parental age effect from phased mutations. a) Paternal age effect on the number of mutations inherited from the father. b) Maternal age effect on the number of mutations inherited from the mother. Lines show predicted number of mutations; colored bands show 95% confidence interval.

Paternal age explained 31% of the variation in the number of DNMs of paternal origin in offspring with a significant effect of 0.6 new mutations each year (slope = 0.64; SD = 0.2; *z* = 3; *P* = 0.002; Fig. 3a). Maternal age explained 22% of the variation, with an increase of 0.08 mutations per year (slope = 0.08; SD = 0.04; *z* = 1.7; *P* = 0.08; Fig. 3b) but the slope did not differ significantly from 0. Both estimates correspond to an average CG of 805 Mb and refer to successfully phased mutations which are a subset of the total. The models mentioned above use either the known age of the parents or an estimate of a minimum age using first observed reproduction (see Materials and Methods). Models using only individuals of known age can be found in Fig. S4.

## Discussion

Using 33 parent–offspring trios we directly estimated the rate of DNMs in the barn owl (*T. alba*) as 4.96 × 10^−9^. This rate is consistent with other passerine species with similar generation times (*g*) of ∼2.5 years such as the zebra finch (*Taeniopygia castanotis*; *g* = 2 to 3 years, *μ* = 5 × 10^−9^; Prentout et al. 2025; *μ* = 6.3 × 10^−9^; Liang et al. 2024), great reed warbler (*Acrocephalus arundinaceus*, *g* ∼ 2.4 years, *μ* = 7.6 × 10^−9^; Zhang et al. 2023), and collared flycatcher (*Ficedula albicollis*, *g* ∼ 2 years, *μ* = 4.6 × 10^−9^; Smeds et al. 2016). While direct comparisons among studies may be complicated by methodological differences (Yoder and Tiley 2021; Bergeron et al. 2022), these results reinforce the substantial dependence of mutation rates on generation times and highlight the comparatively low estimated rate of mutation (9.79 × 10^−10^) in the snowy owl (Bergeron et al. 2023). This discrepancy could be explained by biological differences between the two species. A difference in metabolic rates might impact free radical production and the rate of mutagenesis though in a different direction than the one observed. Studies show that snowy owls consume approximately three times as many kcal as barn owls in a day (Gessaman 1972; Wallick and Barrett 1976). At the same time, the opportunistic breeding of the species through their choice to skip one or more reproductive seasons, might lead to variation in gamete turnover, at least in males, which could alter the rate of mutations per generation. Alternatively, nonbiological factors may also play a role, such as the use of captive individuals which might exhibit a different generation time and are under different stress factors, the use of a single trio of snowy owls, and the completeness of the reference genome or methodological differences in the pipelines used. Using a larger snowy owl dataset from the wild, may help to determine if these differences are due to true underlying factors or an artifact.

The mutational spectrum we identified can be generated by common mutational processes which act across taxa. The most common type of DNMs we found were changes of CpG to TpG which can occur spontaneously through de-amination when the cytosine is methylated (Cooper and Krawczak 1989). Because most cytosines in a CpG context are methylated outside of CpG islands, the dinucleotide shows an increased rate toward TpG. The effect is more pronounced when the DNA is found in single-strand conformation, for example in replication and transcription bubbles and around double-strand breaks (Lujan et al. 2016; Hinch et al. 2023; Williams et al. 2023). Further, this mutational signature seems to accumulate with age (Spisak et al. 2024) and similar signatures have been found across the tree of life (Campbell et al. 2026). In humans (Rahbari et al. 2016) and finches (Prentout et al. 2025), two mutational signatures make up most of the observed spectrum in the germline. These include the aforementioned deamination of CpG > TpG and a wide-ranging signature with the hallmark increase in T > C mutations (second largest class in our dataset). However, decomposition of mutation spectra into mutational signatures requires either a large set of observed DNMs or the use of very rare variants in extensive sequenced datasets, assumed to be very recent and thus influenced only by mutational pressure (Schaibley et al. 2013). With the more limited spectrum presented here (seven classes) we observed few differences among species, suggesting a conservation of underlying processes. On the one hand this is not surprising given that the forces of endogenous (replication fidelity) and exogenous (DNA lesions) mutagenesis along with repair mechanisms are conserved among species. On the contrary, there is evidence of fine-scale differences in mutation spectra of human populations (Harris 2015; Mathieson and Reich 2017; Narasimhan et al. 2017) and it remains unclear how much of the observed variation among species, or absence thereof, is due to methodological differences, stochasticity or a true signal.

The two sexes differ in the number of mutations they pass on to the next generation. We found that males pass on twice as many mutations as females, a male bias of 2.5, one of the lowest estimates for birds. Male bias is present in most species where spermatogonia divide continuously (de Manuel et al. 2022) as errors accumulate with the rate of mitotic division. In accordance, the male bias is notably lower or absent in seasonally breeding fish where sperm cell production is limited to the mating season (Bergeron et al. 2023; Zhang et al. 2023). Nevertheless, there is no consensus if male bias is a byproduct of the proportion of errors that arise, either due to replication, external damage, or the capacity of the male and female germline to repair such errors (Spisak et al. 2024). Since both the frequency of errors and repair efficiency are influenced by epigenetic state, transcriptional activity, and the process of recombination, the rate (and type) of mutations in each sex will reflect the combined effects of sex-specific differences in all these fundamental processes during germline fate (Agarwal and Przeworski 2019; Halldorsson et al. 2019; Hinch et al. 2023; Wyman et al. 2025). As an extension, variation in development of the germline (e.g. methylation state) and life-history traits (e.g. sperm competition and cell divisions in each life stage), will impact the evolution of male bias across species. Thus, the cause of mutational male bias involves multiple interlinked processes covering developmental, molecular, and evolutionary biology.

In addition to the number of mutations they pass on, we also found that males and females differed in the rate of C > T transitions and the rate of CpG > TpG transitions. An increase in C > T transitions in females is also found in humans (Goldmann et al. 2016; Jónsson et al. 2017), suggesting conserved differences between sexes either in the origin of mutations or the repair mechanisms employed. In contrast, sex differences in CpG > TpG transitions have not been described in another species. It is known that CpG dinucleotides undergo spontaneous deamination, an occurrence which correlates strongly and positively with methylation status (Seplyarskiy and Sunyaev 2021). In humans, the proportion of CpG > TpG transitions is higher in younger females compared to younger males (Jónsson et al. 2017). If the same is true in barn owls then it could lead to the observed pattern, as the distribution of female ages in our dataset is skewed toward young individuals (Fig. S5). For example, 27 of the 33 clutches were mothered by females aged 1 or 2 years old (15 of the 33 for males). Alternatively, the difference could be due to a higher overall levels of methylation during oogenesis in birds compared to mammals (Woo and Han 2024). In addition, it could also point to an effect of mutagenic recombination. In humans, recombination leads to an increase of the C to G mutation class in females and of the CpG > TpG in males (Palsson et al. 2025). However, the human-specific C > G enrichment might be linked to the decade-long meiotic stasis of female oocytes as it also scales with maternal age (Jónsson et al. 2017). In addition, bird recombination is localized in CpG islands which might increase the opportunity for CpG > TpG transitions (Singhal et al. 2015). While the mutagenicity of recombination has not been directly demonstrated in birds (but see Rousselle et al. 2019), it is not an unreasonable expectation especially for CpG > TpG transitions which are especially vulnerable at a single-strand conformation as evidenced by their strand asymmetry around crossovers (Lindahl 1993; Halldorsson et al. 2019; Palsson et al. 2025). Ideally, these patterns should be verified with a larger dataset of phased mutations from an avian species before concrete conclusions are reached.

The effect of age on the number of mutations was only significant in males, highlighting that paternal age is a key factor of mutational input in birds, mirroring what has already been observed in mammals (Kong et al. 2012; Goldmann et al. 2016; Rahbari et al. 2016; Jónsson et al. 2017). In contrast, while present, the effect of maternal age was not significant, which may be a result of small sample size and few observations of older mothers as was the case in earlier human studies when the number of trios was limited (Kong et al. 2012; Rahbari et al. 2016). The parental age effect demonstrated here has important practical implications for avian studies using trio-based mutation rate estimates. In datasets where parental ages are unknown or variable, failure to account for age heterogeneity can introduce substantial variance into mutation rate estimates. In addition, a varying age of the parental cohorts might influence the sex bias inferred since families with older fathers than mothers will exhibit stronger male bias, and vice versa. Similarly, if the mutation spectrum changes with age as in humans (Jónsson et al. 2017; Goldmann et al. 2018), the mutational footprint in each sex, and overall, will also depend on the age of individuals sampled. However, the rate of accumulation of new mutations with paternal age represents only a part of the puzzle as it measures the mutation accumulation post sexual maturity (Ségurel et al. 2014; Rahbari et al. 2016). Instead a complete picture will require disentangling the relative rates of errors in each life stage along with knowledge of the number of cell divisions in the germline. Lastly, in many bird species, reproductive performance increases with age (Forslund and Pärt 1995). As a consequence, older males will tend to produce more offspring despite passing on more mutations, a load that could have direct consequences on the fitness of their offspring (Schroeder et al. 2015) and result in a tradeoff between life-history traits (Monaghan and Metcalfe 2019).

## Conclusion

While differences in avian mutation rates and types might be discovered as datasets accumulate, we find this rate in barn owls to be consistent with previous estimates. Species with similar generation times experience similar rates of mutations, suggesting invariable developmental parameters, rates of errors, and efficiency of repair. Further we find that mutations originating in paternal germline increased with age.

## Materials and Methods

### Sample Selection

A set of 57 individuals (31 males and 26 females) in 33 parent–offspring trios was extracted from a large, monitored population of barn owls from Western Switzerland, for which a field-observation pedigree verified by genomic kinship is available (Topaloudis et al. 2025). The choice of individuals was made based on previously available sequences, known age at reproduction and number of offspring of the parents.

### Sequencing and Variant Discovery

The DNA of the 57 individuals was obtained from a blood sample taken from the brachial vein, then immediately frozen in liquid nitrogen and stored in a freezer at −80 °C until DNA extraction. The DNA was then extracted using the DNeasy Blood & Tissue kit (QIAGEN), quantified using the dsDNA HS Qubit kit or the Quant-it PicoGreen dsDNA Assay kit (Thermo Fisher), and diluted to 6.3 ng/μL with 10 mM Tris–HCl, pH 8.0, in 40 μL for Nextera library preparation and to 1.5 ng/μL in 20 μL for Low-Plex library preparation.

All 57 individuals were previously sequenced (Topaloudis et al. 2025). Of these, 52 were resequenced to increase the coverage. When possible, we used the same libraries (46 individuals) and generated new libraries from the same DNA extraction for the rest of the samples (six individuals).

The libraries of 19 samples were previously prepared with PlexWell by Seqwell and sequencing was performed in Illumina NovaSeq 6000. The rest of the libraries (33) were previously prepared with Nextera and sequencing was performed in Illumina HiSeq 4000. For 6 of the 19 Plexwell individuals the old libraries could not be resequenced and new libraries were prepared from a new dilution of the same DNA extraction this time using the Nextera library preparation protocol. All new sequencing data come from three lanes of a NovaSeq X Illumina sequencing machine. Library preparation and sequencing were handled by the Lausanne Genomics Technology Facility.

Newly acquired paired-end sequences of 150 bp were trimmed to a minimum length of 70 bp and adapters were removed using trimmomatic v0.39 (Bolger et al. 2014). Reads were aligned to the barn owl reference genome v4 (Machado et al. 2022) using BWA-MEM v0.7.17 (Li 2013 May 26). Read groups were added using samtools v.1.19.2 (Danecek et al. 2021). Duplicates were marked using GATK v4.5.0.0 (Auwera et al. 2013) and *bam* files were merged with previous sequencing data for the same sample. Base quality score recalibration as suggested by GATK “best practices” was performed using a previously published “true” set of SNPs (Cumer et al. 2022). GATK's *HaplotypeCaller* ran for each sample in the barn owl assembly with default parameters and *-ERC GVCF*. Resulting *gvcf* files were merged per trio using GATK's *CombineGVCFs* with the parameter (*–convert-to-base-pair-resolution*) which resulted in a file with information for every position in the reference. Variant calling used GATK's *GenotypeGVCFs* with –*include-non-variant-sites* enabled.

### Filtering

The following filters and steps were applied per trio, in the order presented, to identify putative DNMs in the autosomal genome (39 linkage groups):

- Regions of the genome with poor mappability, annotated repeats, or around an insertion/deletion polymorphism (indel) were masked. A mappability mask (Corval et al. 2023) removed regions of the genome where less than 95% of the reference fragments (windows of 150 bp, step 1 bp) did not map uniquely and perfectly. In addition, we masked all sites with an annotated repeat or sites that lie closer than 5 bp on either side of the start and end of an indel in each trio.
- Any variant site with genotype quality (GQ) less than 60 and any nonvariant site with a reference genotype quality (RGQ) less than 60 were filtered out.
- For each sample the mean ($\hat{\mu }$) and standard deviation ($\hat{\sigma }$) of read depth was estimated for variant sites. Any position (variant or not) was removed if it had a read depth less than the larger of 15 or $\hat{\mu }\;-2\times \hat{\sigma }\;$. In addition, positions were removed if they had a read depth larger than $2\times \hat{\mu }$ or $\hat{\mu }\;+2\times \hat{\sigma }\;$, whichever value was smaller.
  Since all filters above apply to variant and nonvariant sites, the remaining sequence is the CG of each trio (see below). The following filters apply only to variant sites and need to be accounted for when estimating the FDR (*β*).
- A modified version of the filters suggested in GATK “best practices” was applied (hereafter technical filters). The threshold values were adjusted to be more stringent, as the original ones are lenient by design. Sites were excluded if they failed the following: quality by depth < 5 or mapping quality < 50 or Fisher strand > 20 or strand odds ratio > 2 or mapping quality rank sum test < −4 or read position rank sum test (ReadPosRankSum) < −4.
- Mendelian incompatibilities (MIs) were extracted.
- MIs were selected where the parents were pure homozygotes (no reads supporting the nonreference allele) and the offspring was a heterozygote. Sites were considered as putative mutations if the offspring had an allelic balance (AB; the ratio of reads supporting the alternative allele over the total number of reads at the site) between 0.3 and 0.7. We chose this cutoff, since a mutation inherited from the parents is expected to be found in 50% of the reads (AB = 0.5) with some sampling noise due to sequencing depth and artifacts.

Most estimates of mutation rates focus on sites where both parents are homozygous for the reference allele, assumed to be ancestral, and the offspring is a heterozygote. However, we also included sites where both parents were homozygous for the alternative allele and the offspring a heterozygote. We adopted this approach because the reference genome sample came from our study system and the reference allele cannot be assumed to be ancestral for all trios.

### Estimating Mutation Rate

We defined the mutation rate (*μ*) for each trio following (Bergeron et al. 2021, 2022; Milhaven et al. 2025 Mar 13) as:


$$\mu =\frac{N_{m}}{(1-\beta )\;\times \;\mathrm{CG}\;\times \;2\;}$$

where *N*_m_ is the number of putative DNMs after manual inspection, *β* is the FDR, and CG refers to the callable genome, the size of the genome where mutations could be discovered. We estimated each parameter as follows:

- The number of putative DNMs *N*_m_ was estimated as the number of variants remaining after all filters described above (1 to 6) were applied. To verify these putative mutations we manually inspected each one in the Integrated Genome Viewer v2.19.5 (Robinson et al. 2023) following (Keightley et al. 2014; Wang et al. 2023). For each putative mutation we visualized 2 kb around the variant and examined if reads spanning the mutation also contained other alleles not present in the parents in complete linkage disequilibrium. This would suggest a case of missmapping and we labeled such sites as false positives. We also investigated whether parental reads, before GATK's local realigner, supported the presence of the allele classified as a DNM. From 536 putative forward (reference to alternative allele) and 44 putative reverse (alternative to reference) after manual inspection we retained 280 forward and 4 reverse mutations as true mutational events. For two true DNMs we observed incomplete linkage with an inherited heterozygote nearby, a mosaic pattern, implying a mutation during early development of the offspring. These two sites were considered false positives, when including them, the mutation rate was increased to 4.99 × 10^−9^. We further validated these mutations by testing their presence in a previous sequencing of 346 barn owls from the same population. We removed any mutation which showed presence in individuals either born before the focal individual or born after but with no observed chain of P–O links, or genomic kinship linking the two. Of the 284 mutations, 258 remained, 255 forward and 3 reverse.
- The CG is the number of sites where both parents are homozygotes. These sites were counted after filters one to three were applied (see above). Since all filters up to that step apply to variant and nonvariant sites no correction needs to be applied for an FDR. It has been pointed out that the nonvariant metric of RGQ is not exactly equivalent to GQ but it is not clear how important the discrepancy is (Pfeifer 2021). We assumed the effect to be negligible.

We estimated FDR with an approach adapted from (Bergeron et al. 2021, 2023). The approach attempts to account for filters only applied to variant sites because the metrics are not defined for nonvariant positions (e.g. allelic balance is only defined in heterozygote genotypes). These filters include the GATK technical filters (which often require two alleles) and the AB thresholds of 0.3 and 0.7. The underlying hypothesis is that one can correct for these filters by estimating the proportion of true variants filtered out during these steps. For both cases the “true variants” were defined as sites where parents were pure homozygotes for different alleles and the child an inferred heterozygote (depth for each allele > 0). The proportions of these “true mutations” removed after applying either the AB or the technical filters was calculated, and the sum of these proportions was used to estimate the FDR. A quantification and decomposition of the final FDR used per trio can be found in Fig. S6.

### Sex Chromosome

We followed the same approach for the Z chromosome with a key difference. We treated the pseudoautosomal region (PAR) (Topaloudis et al. 2025), and the rest of the chromosome (non-PAR) in two different ways. SNP-calling, filtering and mutation identification in the PAR region was applied exactly as described for autosomal regions. In the non-PAR, females were called as haploids and when applying an individual depth filter in females we halved both the maximum and minimum depth thresholds. For mutation identification in haploid females we did not filter on AB and asked instead that 0 reads supported the reference allele. When estimating the mutation rate in the Z chromosome, we used the following approach to account for the single copy non-PAR in females (multiplied by 3/2 instead of 2):


$$\mu =\frac{N_{m}}{\left(1-\beta )\;\times \;(3/2\;\times \;\mathrm{nonPAR}\;+\;2\;\times \;\mathrm{PAR}\right)}$$

### Phasing De Novo Mutations

DNMs were phased by read-based phasing of the proband using WhatHap v.1.6 (Martin et al. 2016). When the DNM was placed in a phase set (i.e. was phased with other nearby heterozygous variants), we looked for phase-informative variants in the phase set to identify paternal or maternal inheritance. Phase-informative sites are defined as sites where the proband is heterozygous and at least one of their parents is not.

When possible, (nine individuals), we phased mutations by transmission to a third generation. Phase-informative sites (defined as above) were identified in a region of 10 kb around the DNM and grand-paternal origin was inferred in the offspring of the proband. Segregation to a third generation was also used to test the Mendelian inheritance of detected DNMs using a binomial test (*binom.test* function in R) of the observed genotypes of the third generation at the mutated site (0, not inherited; 1, inherited).

### Mutation Spectrum

For each mutation we extracted the two adjacent nucleotides from the reference sequence using bedtools2 (Quinlan 2014). Mutations were classified according to the nucleotide change that occurred and reverse complements were collapsed (e.g. C > T and G > A). Mutations of the C to T nature occurring in CpG dinucleotides were separated into their own class. The full trinucleotide spectrum can be found in Fig. S7 For sex-specific spectra we used the same process but with only the phased mutations coming from each sex. To compare the sex spectra we used Fisher's exact test implemented using *fisher.test* in R with simulate.p.value = TRUE and *B* = 100,000 (number of simulations). Multiple testing correction was implemented with p.adjust using the Benjamini and Hochberg method (method = “BH”) (Benjamini and Hochberg 1995).

### Age Effect

Using a generalized linear mixed model with the *glmmTMB* function of glmmTMB package v1.1.13 (Brooks et al. 2025), we regressed the phased mutations originating from each sex to the age of the parent of origin (in years), fitting the trio's CG size (in Gb or 10^9^ bp) as a fixed effect. We build a separate model for each sex. The birth year of individuals born in one of the monitored nestboxes is known, and so is their age at reproduction (8/16 fathers, 7/17 mothers). For the rest of the samples, we can estimate a minimum age by assuming the individual was born at least 1 year before its first reproductive attempt because barn owls start reproducing as yearlings. Using a generalized linear mixed model, we regressed the inferred phased mutations from each parent to their estimated age. Male mutations were modeled using a Poisson distribution with an identity link and the parent's id as a random effect. Female mutations were modeled using a zero-inflated Poisson with a log link, to account for a large number of families with no phased mutations of female origin. Including zero inflation or not returned almost identical results. Model fitting was tested with DHARMa v0.4.7 (Hartig 2024). We also ran the same models using only the true age of individuals born in the system.

Generation time was estimated as the average age of reproducing individuals in the system (Fig. S8). All statistical analyses and figures were generated using R v4.5.1 (R Core Team 2025) with the help of *tidyverse* v2.0.0 (Wickham et al. 2019), *ggpubr* v0.6.1 (Kassambara 2025), and *viridis* v0.6.5 (Garnier et al. 2024).

## Supplementary Material

evag175_Supplementary_Data

## Data Availability

All old sequencing data can be found on the Sequence Read Archive of NCBI under Bioproject PRJNA1172395 and new ones under PRJNA1475960. Scripts with all analyses performed can be found on https://github.com/topalw/Mutations and a stable version with intermediate data can be found on Zenodo under https://doi.org/10.5281/zenodo.21076869.
