# Supplementary material for: The Effect of Age and Sex on the Rate of Germline Mutations in Barn Owls
Source: Genome Biol Evol. 2026 Jul 10;18(7):evag175. doi: 10.1093/gbe/evag175 (PMC13399160; doi:10.1093/gbe/evag175)
Supplement: evag175_Supplementary_Data [file evag175_supplementary_data.zip › Supplementary_Material_v2.pdf]

Supplementary Material

Supplementary Material **1**
Supplementary Figures 2

Supplementary Tables

Supplementary Table 2. A comparison of datasets, including filters used in the different studies compared in Supplementary Figure 3.

Filter / Attribute	This study	Great reed warbler (Zhang et al., 2023)	Collared flycatcher (Smeds et al., 2016)	Zebra finch (Prentout et al., 2025)	Humans (Rahbari et al., 2016)
Number of samples	57	12	11	74	19 (new)
Number of trios	33	16	7	40	12 (new)<br>109 (compiled)
Number of mutations	284	82	55	202	768 (new)<br>6570 (compiled)
Average sequencing depth	54x	41x	42x	25x	24.7x
Trimming	Trimmomatic	Trimmomatic	-	cutadapt	-
Mapping	bwa-mem	bwa-mem	BWA	bwa-mem	-
Variant calling	GATK	freebayes	GATK	GATK (through sarek)	DeNovoGear
Mappability	Mappability mask (snpable) 95% cutoff	Annotated repeats excluded	Annotated repeats excluded	Mappability mask (snpable) 100% cutoff	Removed low-complexity regions
INDELs	Excluded 5bp around	removed	removed	Excluded 5bp around	-
Filters	GQ > 60<br>QD > 5<br>MQ > 50	QUAL > 30<br>SAF > 0<br>SAR > 0	GQ >= 30<br>.25 < AB	GQ >= 30<br>2 reads with alternative	5% of reads supporting alternative

	FS < 20 SOR < 2 MQRankSum > -4 ReadPosRankSum > -4	RPR > 0 RPL > 0 GT/DP[*] > 9		allele in proband	allele in parents
DP	max(15, mean - 2 x sd) to min(2 x mean, mean + 2 x sd)	.5 to 1.5 x median	>= 15	8 to 2 x mean	Remove top 0.01% quantile
CG	Filter non-variant sites	Depth filter only	Depth filter + no repeats + callable loci (GATK)	Depth filter + indel filter + mappability + no repeats	>= 7x reads + repeats
FPR	Manual inspection + inheritance + pop sample	inheritance	Manual inspection + inheritance + pop sample + resequencing	Manual curation + pop sample	resequencing
FDR approach	Proportion of AA x BB = AB filtered out	Simulation of mutations	SNP genotyping	-	resequencing

Supplementary Figures

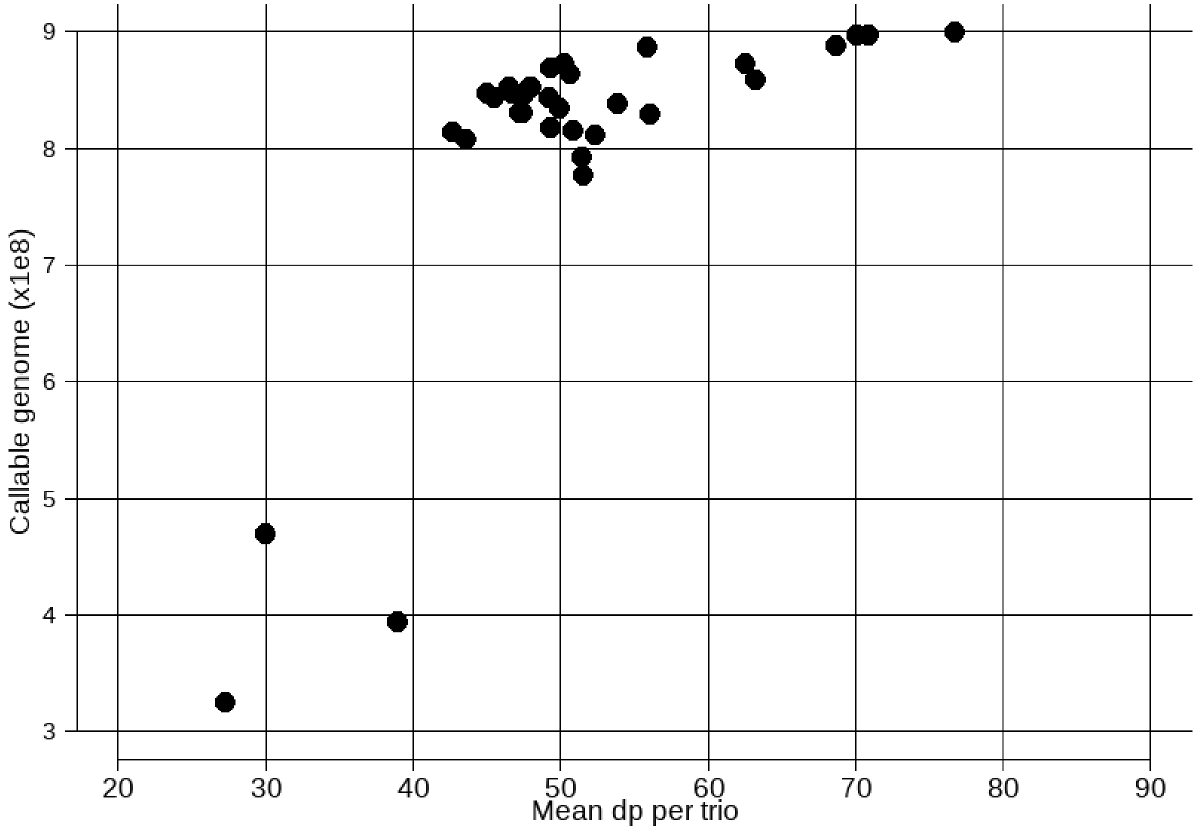

Supplementary Figure 1. The callable genome(bp) in each trio as a function of average sequencing depth.

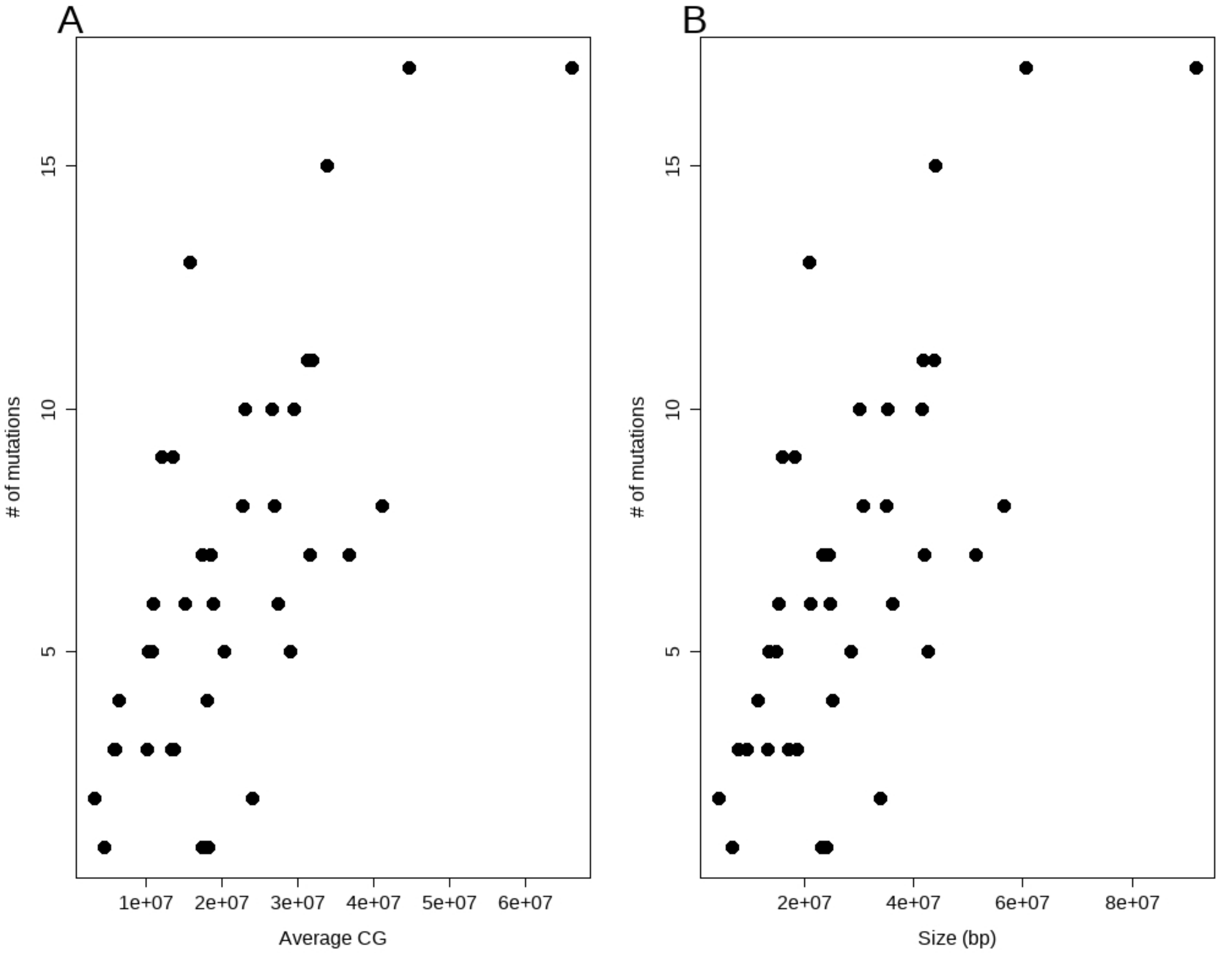

Supplementary Figure 2. The number of mutations as a function of average callable genome per linkage group (A) or physical length (B).

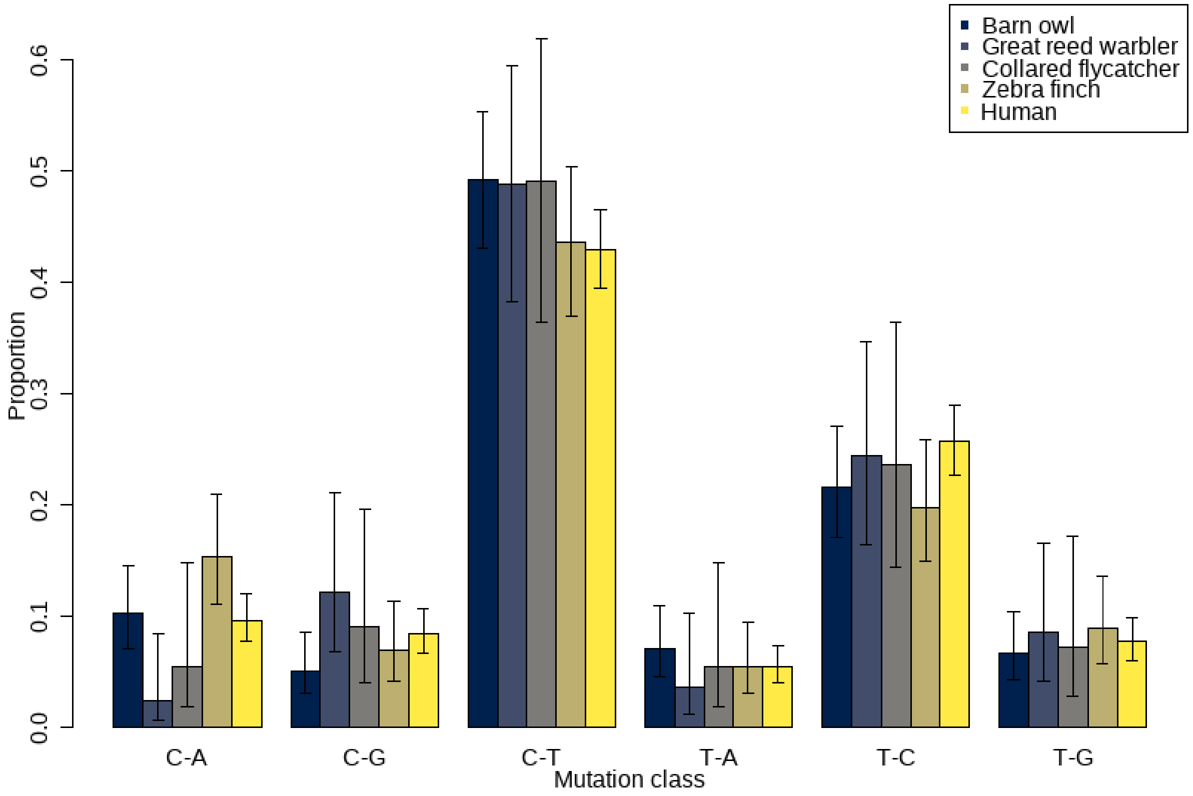

Supplementary Figure 3. The low resolution mutation spectrum across studies. Because some studies did not specify CpG dinucleotides the C>T class contains all C>T mutations. Origin of data: Great reed warbler - (Zhang et al. 2023); Collared flycatcher - (Smeds et al. 2016); Zebra finch (Prentout et al. 2025); Human - (Rahbari et al. 2016). Error bars show 95% binomial confidence intervals.

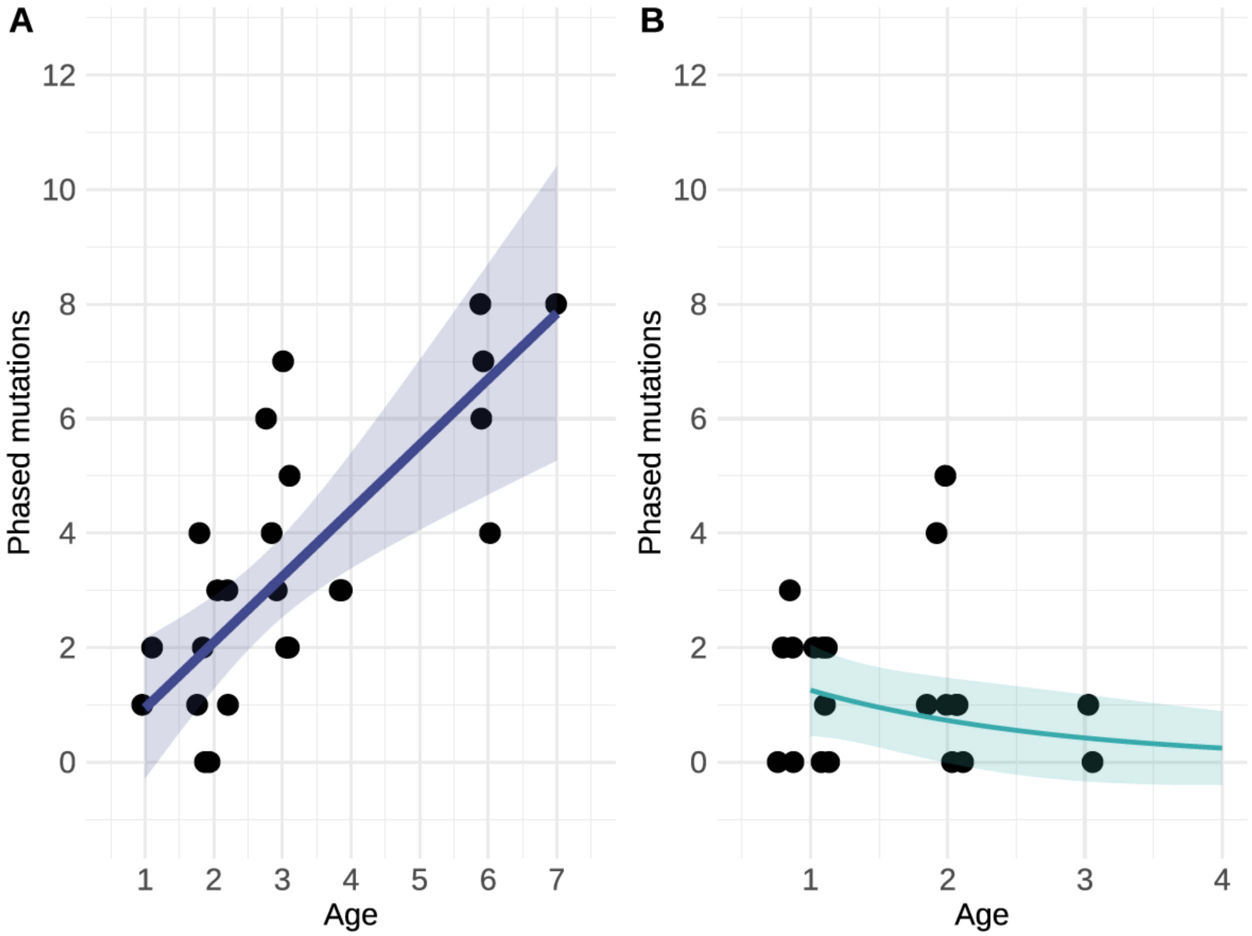

Supplementary Figure 4. The age effect using only individuals of known age. Models as in the main text. Left males (slope = 1.14; z = 3.68; $p < 0.001$); Right females (slope = -0.56; z = -0.95; p = 0.34)

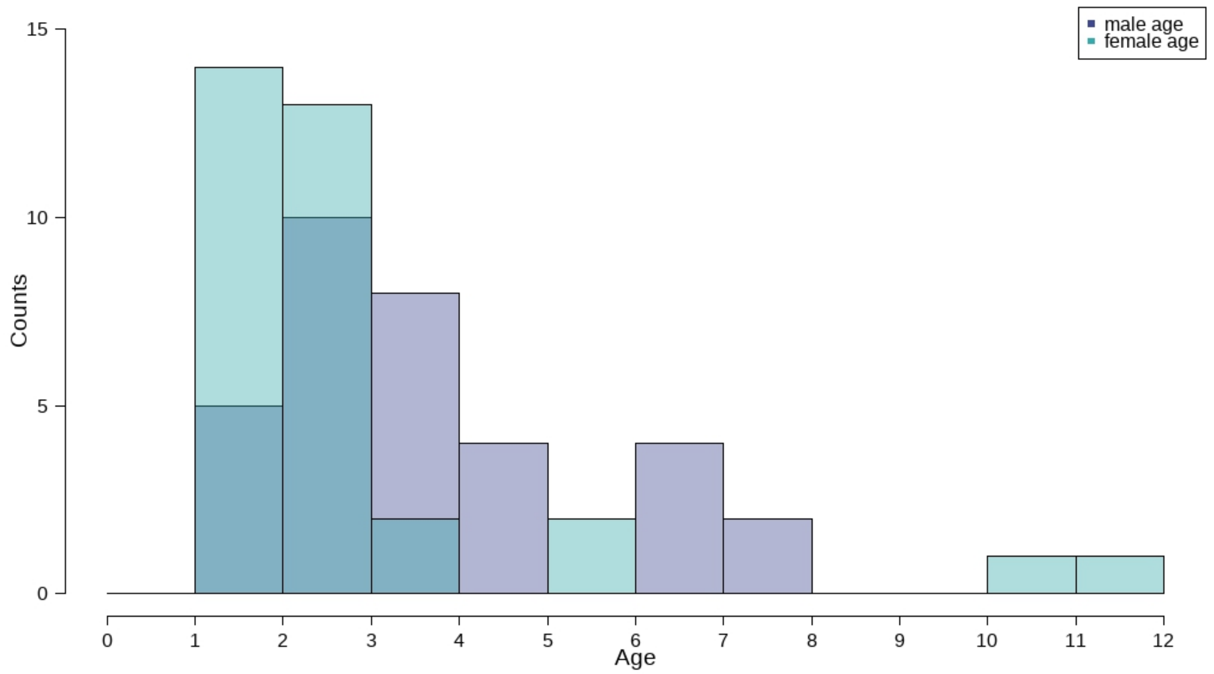

Supplementary Figure 5. The distribution of age at reproduction in the trios used.

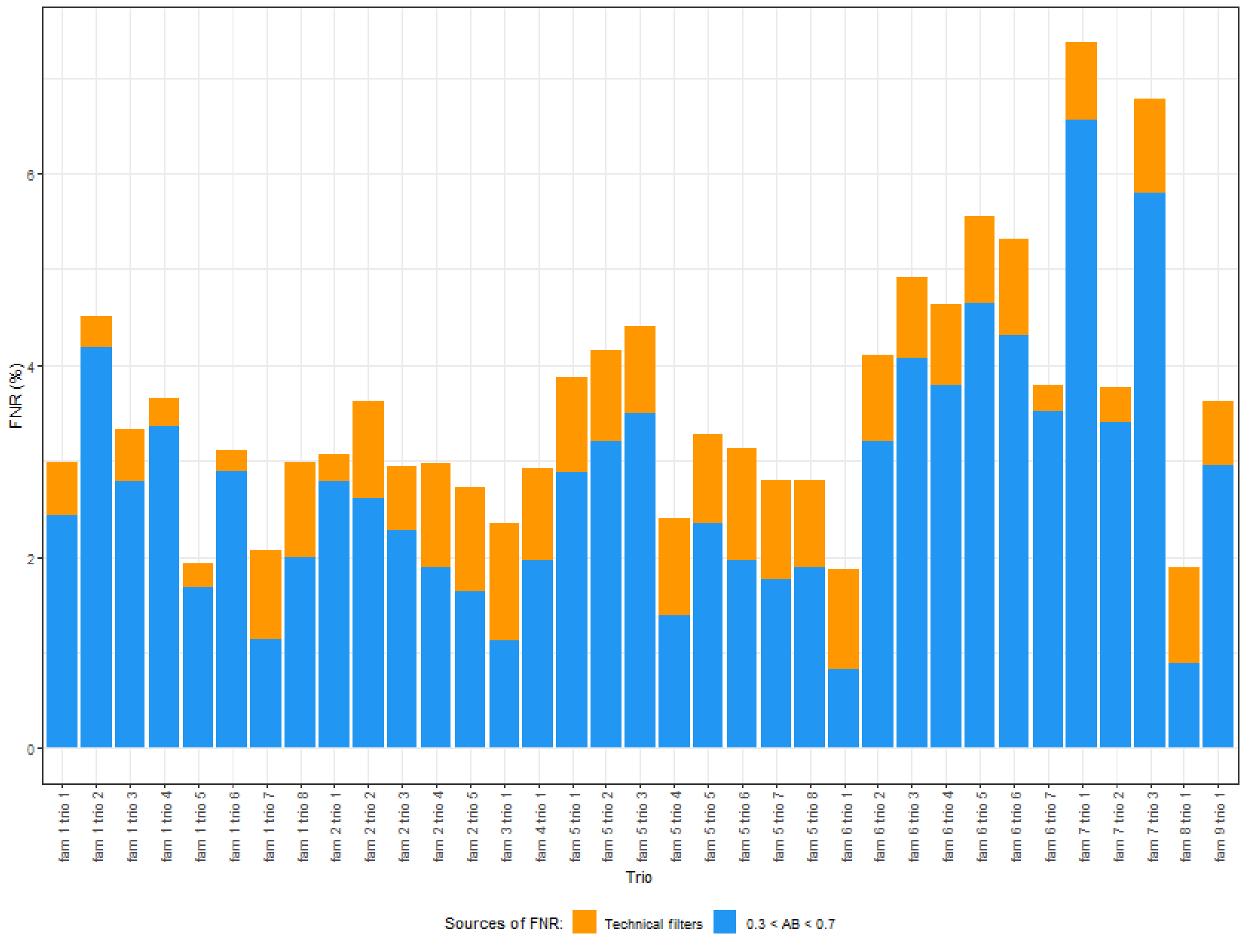

Supplementary Figure 6. The total False Negative Rate (also False discovery rate - FDR) estimated in the study for each trio. The proportion can be due to the Allelic Balance filter (blue bars) or the GATK technical filters applied. It is estimated as the proportion of ‘true’ heterozygous sites in the offspring (parents homozygous for opposite alleles), filtered out due to each filter.

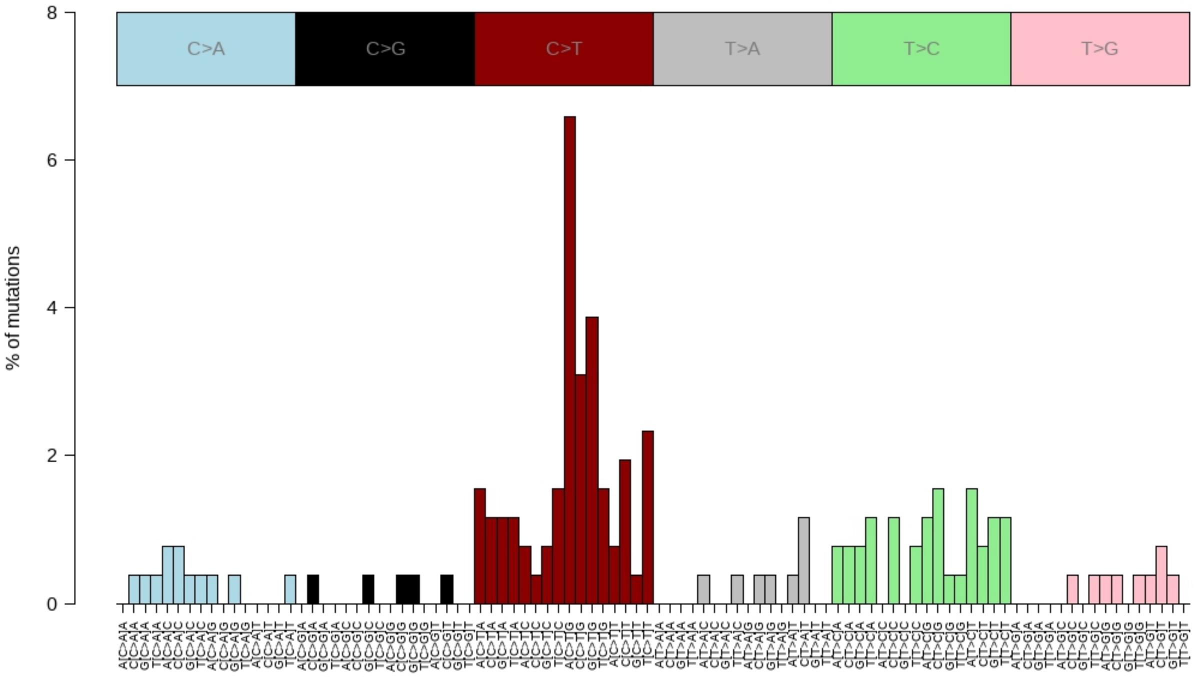

Supplementary Figure 7. The high resolution mutation spectrum for our dataset.

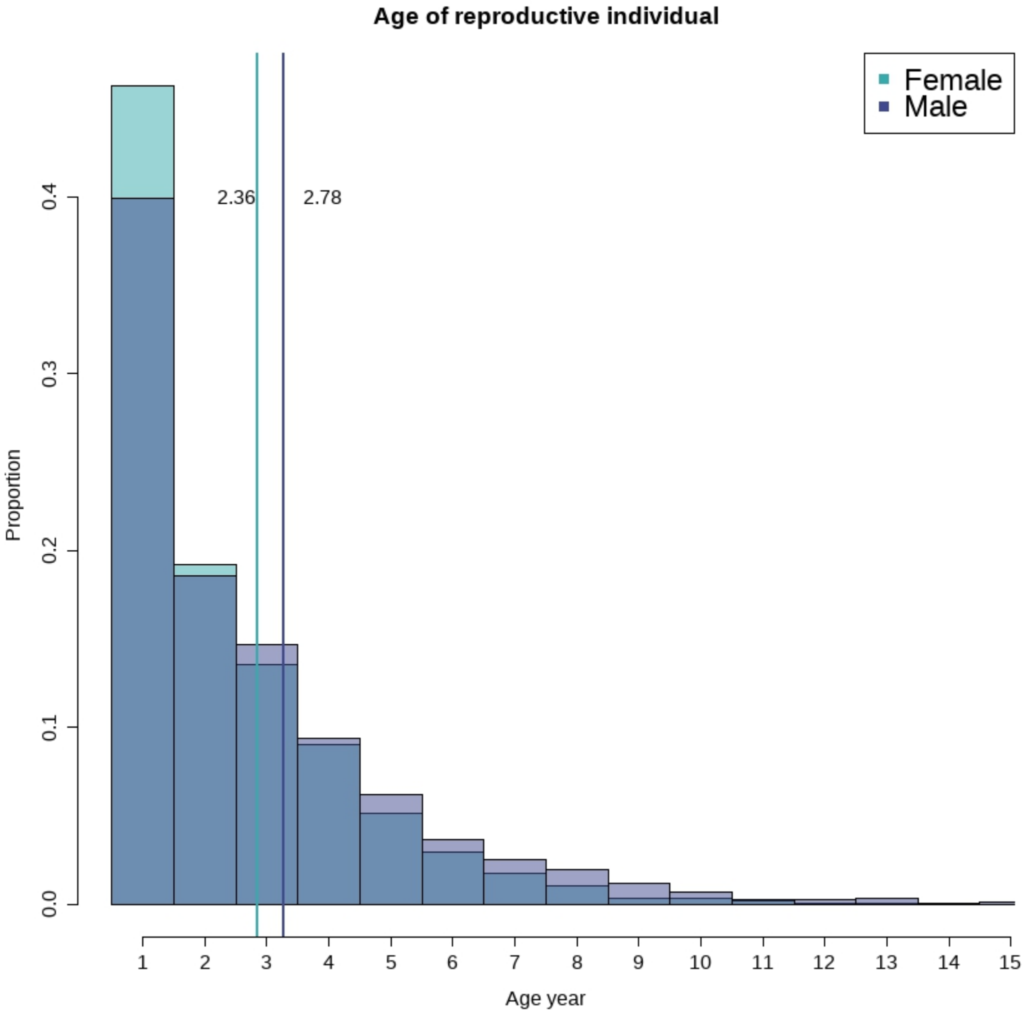

Supplementary Figure 8. Average age of individuals reproducing in the long term study. The average age between the sexes is 2.57 years.
